# Enhancement of Antigen Presentation by Deletion of Viral Immune Evasion Genes Prevents Lethal Cytomegalovirus Disease in Minor Histocompatibility Antigen-Mismatched Hematopoietic Cell Transplantation

**DOI:** 10.3389/fcimb.2020.00279

**Published:** 2020-06-09

**Authors:** Emin Gezinir, Jürgen Podlech, Kerstin M. Gergely, Sara Becker, Matthias J. Reddehase, Niels A. W. Lemmermann

**Affiliations:** Institute for Virology and Research Center for Immunotherapy (FZI) at the University Medical Center of the Johannes Gutenberg-University of Mainz, Mainz, Germany

**Keywords:** bone marrow transplantation, CD8 T cells, graft-vs.-host disease (GvHD), hematopoietic reconstitution, minor histocompatibility antigens, murine cytomegalovirus, nodular inflammatory focus (NIF), transplantation tolerance

## Abstract

Hematoablative treatment followed by hematopoietic cell transplantation (HCT) for reconstituting the co-ablated immune system is a therapeutic option to cure aggressive forms of hematopoietic malignancies. In cases of family donors or unrelated donors, immunogenetic mismatches in major histocompatibility complex (MHC) and/or minor histocompatibility (minor-H) loci are unavoidable and bear a risk of graft-vs.-host reaction and disease (GvHR/D). Transient immunodeficiency inherent to the HCT protocol favors a productive reactivation of latent cytomegalovirus (CMV) that can result in multiple-organ CMV disease. In addition, there exists evidence from a mouse model of MHC class-I-mismatched GvH-HCT to propose that mismatches interfere with an efficient reconstitution of antiviral immunity. Here we used a mouse model of MHC-matched HCT with C57BL/6 donors and MHC-congenic BALB.B recipients that only differ in polymorphic autosomal background genes, including minor-H loci coding for minor-H antigens (minor-HAg). Minor-HAg mismatch is found to promote lethal CMV disease in absence of a detectable GvH response to an immunodominant minor-HAg, the *H60* locus-encoded antigenic peptide LYL8. Lethality of infection correlates with inefficient reconstitution of viral epitope-specific CD8^+^ T cells. Notably, lethality is prevented and control of cytopathogenic infection is restored when viral antigen presentation is enhanced by deletion of immune evasion genes from the infecting virus. We hypothesize that any kind of mismatch in GvH-HCT can induce “non-cognate transplantation tolerance” that dampens not only a mismatch-specific GvH response, which is beneficial, but adversely affects also responses to mismatch-unrelated antigens, such as CMV antigens in the specific case, with the consequence of lethal CMV disease.

## Introduction

Hematopoietic cell transplantation (HCT) is the treatment of choice for hematopoietic malignancies that are resistant to standard therapies. In essence, leukemic cells are wiped out by hematoablative procedures, and co-ablated cells of the immune system become reconstituted by HCT. This therapy is inherently associated with a transient immunocompromised state in HCT recipients that lasts until transplanted hematopoietic stem cells have restored mature cells of all hematopoietic differentiation lineages. This opens a “window of opportunity” for latent cytomegalovirus (CMV) to reactivate to productive infection, which is a “window of risk” in medical view. If left untreated, CMV reactivation can result in an unrestrained lytic CMV replication that leads to lethal multiple organ failure, with interstitial pneumonia representing the most feared organ manifestation. Accordingly, follow-up monitoring of HCT recipients for CMV reactivation is routine in all transplantation centers worldwide to initiate pre-emptive antiviral therapy upon first detection (Hebart and Einsele, 2004; Seo and Boeckh, 2013; Stern et al., 2019). In HCT, risk is highest in CMV-antibody seropositive recipients receiving hematopoietic stem and progenitor cells (HC) from CMV-antibody seronegative donors (risk in D^−^R^+^ > risk in D^+^R^−^ constellation), which indicates that CMV reactivates mainly from latently infected tissue cells already present pre-transplantation in recipients' organs (Emery, 1998; for a recent review on latently infected cell types, see Reddehase and Lemmermann, 2019).

The outcome of HCT in general as well as with respect to CMV reactivation is largely influenced by the immunogenetics of donor and recipient. In autologous HCT, HC isolated pre-transplantation from the recipient are re-infused after the hematoablative leukemia therapy, so that missing difference in histocompatibility antigens in all likelihood excludes any complication by graft-vs.-host (GvH) reaction. In syngeneic HCT with identical twins, donor and recipient are genetically identical but epigenetically disparate. So, if gene desilencing in the recipient twin affects a histocompatibility antigen for which the encoding gene remained silenced in the donor twin, a GvH reaction might be triggered. Indeed, cases of GvH disease (GvHD) were reported for recipients of HC from identical twin donors (Rappeport et al., 1979), although in this early report epigenetics was not considered as an explanation.

In allogeneic HCT with family donors or unrelated donors, differences in HLA/MHC antigens and/or minor histocompatibility antigens (minor-HAg) are unavoidable and bear a risk of GvHD. HLA/MHC matching between recipients and unrelated donors is used to minimize disparity in MHC class-I and avoid disparity in MHC class-II, but mismatches in non-MHC polymorphic autosomal background genes, including minor histocompatibility (minor-H) loci coding for minor-HAg, is unavoidable. Specifically, HLA-identical sibling donor and recipient pairs differ in minor-HAg (Bleakley et al., 2004). A pathogenetic link between donor-recipient mismatch in major and/or minor histocompatibility antigens is suggested by early reports showing a higher incidence of CMV organ disease after allogeneic HCT compared to syngeneic (Applebaum et al., 1982; Meyers et al., 1982) or to autologous HCT (Wingard et al., 1988).

As clinical investigation in patients cannot address mechanisms by experimental approaches involving genetically modified viruses with targeted mutations (Lemmermann et al., 2011), tailored mouse models based on murine CMV (mCMV) have been established for studying general principles of pathogenesis and immune control of a CMV in its natural host (reviewed in Reddehase and Lemmermann, 2018). Our previous work was focused on a mouse model of experimental syngeneic HCT and mCMV infection with BALB/c mice as donors and recipients to understand this basal HCT setting before considering the complicating consequences of immunogenetic mismatches (reviewed in Holtappels et al., 2013; Reddehase, 2016). As a key finding, control of mCMV infection in HCT recipients was found to rest upon a timely and also quantitatively efficient hematopoietic reconstitution of antiviral CD8^+^ T cells (Holtappels et al., 1998; Podlech et al., 1998, 2000). Adoptive transfer of mature antiviral CD8^+^ T cells was found to bridge the “window of risk” between the time of HCT and completion of hematopoietic reconstitution. Combining HCT and CD8^+^ T-cell transfer prevented viral pathogenesis in the recipients (Steffens et al., 1998a), including a CMV-associated graft failure caused by inhibition of bone marrow repopulation due to infection of the bone marrow stroma (Mayer et al., 1997; Steffens et al., 1998b; Renzaho et al., 2020).

In recent work (Holtappels et al., 2020), we extended this basal model by introducing a singular MHC class-I disparity between HCT donors and recipients. This disparity is based on a spontaneous deletion of a region encompassing the MHC class-I gene *L*^*d*^ in BALB/c mice, resulting in the congenic mutant mouse strain BALB/c-H-2^dm2^. This special case of a mismatch opened the chance to separate GvH reactivity from host-vs.-graft (HvG) reactivity. Using the mutant strain as donor and the parent strain as recipient defines GvH-HCT, whereas the inverse transplantation direction defines HvG-HCT. The comparison between these two types of HCT revealed a high lethality after mCMV infection selectively in the GvH setting. Notably, lethality could not be attributed to a GvH reaction against the MHC class-I molecule L^d^ expressed in the BALB/c recipients. Thus, the cause of death was not GvHD. Instead, lethal viral pathogenesis resulted from a failure in controlling the infection due to an insufficient reconstitution of high-avidity viral epitope-specific CD8^+^ T cells capable of recognizing infected cells.

Although this model was academically elegant by separating potential GvH and HvG complications of allogeneic HCT, one can see a limitation in the fact that mismatch by genetic deletion of an MHC antigen has no obvious correlate in clinical HCT, as far as we know. As a step bringing the model closer to clinical HCT, we have here avoided this limitation by studying GvH-HCT with MHC-matched unrelated HCT donors and recipients that differ only in polymorphic autosomal minor-H loci.

A well-defined minor-HAg mismatch was introduced into the HCT model by using C57BL/6 (*H-2*^*b*^) mice as donors and MHC congenic BALB.B mice as recipients in a GvH-HCT. Based on their BALB/c genetic background, BALB.B mice express the minor-HAg H60 from the *H60* locus on chromosome 10. This minor-HAg contains the immunodominant naturally processed octapeptide LTFNYRNL (H60-LYL8), which is presented by the MHC class-I molecule K^b^ as a peptide MHC-I (pMHC-I) complex (Malarkannan et al., 1998; Choi et al., 2002a,b). *H60* is a gene family coding for proteins H60a, H60b, and H60c, all of which yield the LYL8 peptide by antigen processing. They differ in their tissue distribution, all sparing the lungs and being poorly expressed in the liver, whereas H60a is highly expressed in the central lymphoid organs thymus and spleen (Takada et al., 2008). As H60 proteins are not expressed from the genetic background of C57BL/6 mice, H60-specific reactivity between the two mouse strains is unidirectional, so that an H60-specific HvG reverse reaction does not occur in this particular minor-HAg GvH-HCT model. Notably, native H60 proteins are ligands of NKG2D, a costimulatory receptor expressed on activated CD8^+^ T cells, so that native H60 proteins and presented H60-LYL8 peptide might synergize in GvH-reactivity (Cerwenka et al., 2002; Takada et al., 2008). To sum this up, from the universe of potential minor-HAg mismatches in this MHC-matched “unrelated donor” GvH-HCT model, H60 stands out because C57BL/6 donors develop an exceedingly high T-cell repertoire dedicated to it (for a review, see Roopenian et al., 2002).

Such an experimental setting does have a correlate in clinical HCT with unrelated donors, where MHC matching is the gold standard. This new model reproduced enhanced lethality of mCMV infection in GvH-HCT compared to syngeneic HCT, and again, lethality was caused by uncontrolled virus replication and consequent histopathology due to an insufficient reconstitution of antiviral CD8^+^ T cells.

## Materials and Methods

### Mouse Strains

Breeding pairs of mouse strain BALB.B (MHC class-I genes *H-2 K*^*b*^*, -D*^*b*^; Freedman and Lilly, 1975) were purchased from the Jackson laboratory (JAX stock #001952). BALB.B and the MHC congenic C57BL/6 (*H-2 K*^*b*^*, -D*^*b*^) mice were bred and housed under specified-pathogen-free conditions at the Central Laboratory Animal Facility of the Johannes Gutenberg University Mainz, Mainz, Germany. Mice were used in experiments at an age of 8–10 weeks.

### Viruses and Cells

Cell culture-derived BAC-free high-titer virus stocks of mCMV-SIINFEKL (here referred to as wild-type virus, WT) and mCMV-ΔvRAP-SIINFEKL (Lemmermann et al., 2010a), briefly ΔvRAP virus, were generated by standard protocol (for method book chapters, see Podlech et al., 2002; Lemmermann et al., 2010b). Murine embryonic fibroblasts (MEF) were prepared from C57BL/6 mice and cultivated as described (Podlech et al., 2002). EL-4 cells (ATCC TIB-39) were cultivated in Dulbecco's Modified Eagle's Medium (DMEM) containing 10% fetal calf serum.

### Generation of Recombinant Virus

The bacterial artificial chromosome (BAC)–derived recombinant virus mCMV-Δm157-SIINFEKL (briefly Δm157 virus) was generated with methods essentially as described previously (Lemmermann et al., 2010a). In brief, two-step BAC mutagenesis was performed by standard protocol (Borst et al., 2004) using shuttle plasmid pST76K-m164_SIINFEKL (Lemmermann et al., 2010a) and BAC plasmid pSM3frΔm157 (Bubic et al., 2004).

### Experimental HCT

HCT was performed as described in greater detail previously (Podlech et al., 2002). In essence, HCT recipient mice were subjected to total-body γ-irradiation with a single dose of 7 Gy. Donor-derived femoral and tibial bone marrow cells (BMC) were immunomagnetically depleted of mature CD4^+^ and CD8^+^ T cells to restrict the immunological analyses in HCT recipients to T cells derived by hematopoietic reconstitution in the T-cell differentiation lineage. Note that undepleted BMC would include 1–2% mature T cells, derived mainly from the BM vasculature. HCT was performed by infusion of 5 × 10^6^ BMC into the tail vein of the recipients. Subsequently, the recipients were infected in the left hind footpad with 1 × 10^5^ plaque-forming units (PFU) of the indicated recombinant mCMVs.

### Antigenic Peptides

Synthetic peptides corresponding to viral epitopes presented by MHC class-I molecules K^b^ and D^b^ are derived from sequences of the mCMV open reading frames (ORFs) *M38, M45, M57, m139*, and *M122/IE3* (for peptide sequences, see Munks et al., 2006). The K^b^-presented octapeptides SIINFEKL (Carbone and Bevan, 1989; Falk et al., 1991; Rötzschke et al., 1991) and LTFNYRNL (LYL8) (Malarkannan et al., 1998) are derived from the sequences of ovalbumin and of the minor-HAg H60, respectively. Custom peptide synthesis with a purity of > 80% was performed by JPT Peptide Technologies (Berlin, Germany).

### Assays of CD8^+^ T-Cell Effector Functions

IFNγ-based enzyme-linked immunospot (ELISpot) assays were used to detect sensitization of CD8^+^ T cells by MHC class-I-presented synthetic antigenic peptides (Pahl-Seibert et al., 2005; Böhm et al., 2008b, and references therein). In brief, graded numbers of immunomagnetically purified CD8^+^ T cells were sensitized in triplicate assay cultures by incubation with EL-4 (*H-2*^*b*^) target cells exogenously loaded with synthetic antigenic peptides at a saturating concentration of 10^−6^ M. Frequencies of IFNγ-secreting cells and the corresponding 95% confidence intervals were calculated by intercept-free linear regression analysis.

### Quantitating *in vivo* Infection and Tissue Infiltration of Reconstituted T Cells

At indicated times post-HCT and infection, the load of infectious virus in spleen, lungs, liver, and salivary glands was determined for the respective organ homogenates by virus plaque assay performed under conditions of “centrifugal enhancement of infectivity” increasing detection sensitivity by a factor of ca. 20 (Kurz et al., 1997; Podlech et al., 2002, and references therein). Infected cells and T cells in liver tissue sections were detected and quantified by two-color immunohistochemistry (2C-IHC) specific for the intranuclear viral IE1 protein (red staining) and the cell membrane T-cell receptor (TCR) complex molecule CD3ε (black staining) as described in greater detail previously (Podlech et al., 2002; Lemmermann et al., 2010b).

### Statistical Analyses

To evaluate statistical significance of differences between two independent sets of log-transformed, log-normally distributed data, the two-sided unpaired *t*-test with Welch's correction of unequal variances was used. In case of data sets that include data below detection limit, which excludes log-transformation, the distribution-free Wilcoxon Mann Whitney test was applied. Differences were considered as statistically significant for *P*-values of < 0.05 (^*^), < 0.01 (^**^), and < 0.001 (^***^). Kaplan-Meier survival plots were used for documenting survival in independent cohorts. Statistical significance of differences between groups was calculated with log-rank and Gehan-Wilcoxon test. Calculations were performed with GraphPad Prism 6.07 (GraphPad Software, San Diego, CA, USA).

Frequencies of IFNγ-secreting cells responding in the ELISpot assay and the corresponding 95% confidence intervals were calculated by intercept-free linear regression analysis based on spot counts from triplicate assay cultures for each of the graded cell numbers seeded, as described previously (Pahl-Seibert et al., 2005; Böhm et al., 2008b). Calculations were performed with Mathematica, 8.0.4 (Wolfram Research, Champaign, Il, USA).

## Results and Discussion

### Viral Immune Evasion Gene-Dependent Lethality of CMV Infection in Minor Histocompatibility Antigen-Mismatched GvH-HCT

In HCT with unrelated donors, MHC/HLA matching between donor and recipient is performed to minimize and ideally avoid MHC/HLA mismatches, in particular MHC class-II mismatches that could induce GvHD mediated by CD4^+^ T cells through a “cytokine storm” without requirement of cognate T-cell interaction with MHC class-II antigen that is anyway not constitutively expressed by tissue cells (for a review, see Reddy et al., 2008). Even after complete MHC antigen match, however, a risk for GvHD results from unavoidable mismatches in polymorphic autosomal loci coding for minor-HAg.

Previous work in the mouse model of GvH-HCT with an MHC class-I gene deletion mismatch has attributed unrestrained viral spread and lethal histopathology to an insufficient reconstitution of high-avidity antiviral CD8^+^ T cells needed for recognizing and attacking infected tissue cells. Notably, GvH-HCT recipients survived infection with a recombinant mCMV in which immune evasion genes were deleted to prevent inhibition of the MHC class-I pathway of antigen presentation in infected tissue cells (Holtappels et al., 2020).

**Figure 1:**
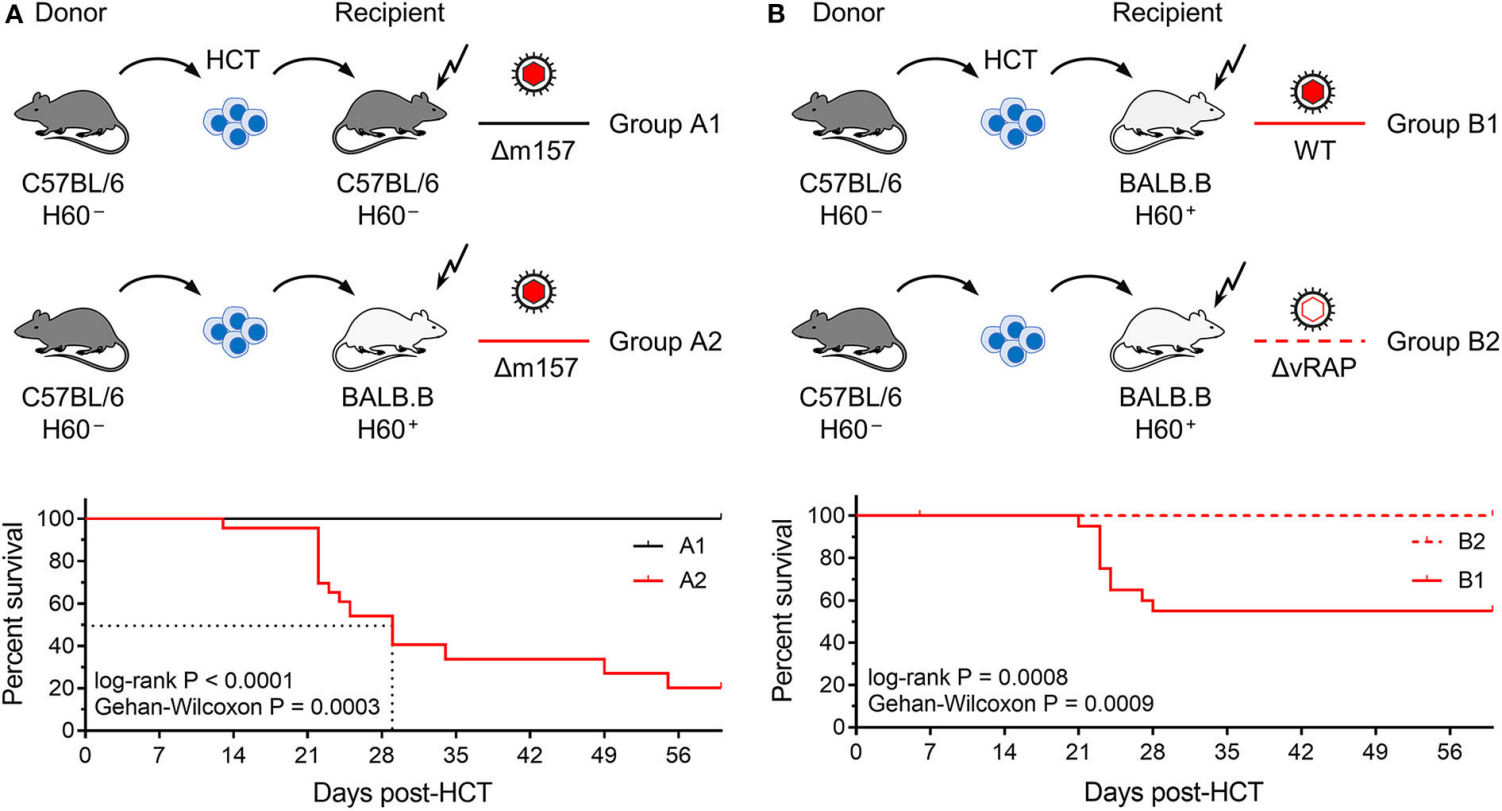
Lethality of infection after syngeneic and minor-HAg H60-mismatched GvH-HCTs. Sketches of the transplantation protocols define in **(A)** the HCT groups A1 and A2 (*n* = 35 recipients each), and in **(B)** the HCT groups B1 and B2 (*n* = 45 recipients each). These groups are studied throughout the manuscript. The flash symbol indicates hematoablative conditioning of the HCT recipients by total-body γ-irradiation. All HCTs were performed with T cell-depleted donor bone marrow cells to exclude GvHD induced by transplanted mature T cells. Viruses used for infection in the different HCT groups are indicated. **(A)** Kaplan-Meyer survival plots comparing group A1 (*n* = 23 recipients, all survived) with group A2 (*n* = 23 recipients, 5 survived). The dotted lines indicate the 50% median survival time, which was 29 days. **(B)** Kaplan-Meyer survival plots comparing group B1 (*n* = 21 recipients, 12 survived) with group B2 (*n* = 21 recipients, all survived). 50% lethality was not reached for calculation of a 50% median survival time.

Here we modeled the clinically relevant case of MHC match and minor-HAg mismatch. Two pairs of two HCTs, each pair tested in one experiment for a direct and unbiased comparison, were performed to study the impact of minor-HAg, including the well-defined H60 antigen, on lethality from mCMV infection (Figure 1). All HCTs share C57BL/6 (MHC class-I and class-II molecules of haplotype *H-2*^*b*^) as donors, so that bone marrow repopulation efficacies of donor hematopoietic stem and progenitor cells as well as the donor T-cell specificity repertoire and donor natural killer (NK) cell subsets are no variables in these HCT settings. Accordingly, all differences in the outcome can be attributed to recipients' immunogenetics and to virus genetics.

In the first pair of HCTs (Figure 1A), syngeneic HCT with C57BL/6 mice as donors and recipients (group A1) was tested as a reference system with identity in genetically determined MHC antigens as well as minor-HAg, while not excluding epigenetic mismatches. Epigenetic differences are leveled out by using pools of donor HC, but may nevertheless contribute to the variance seen between individually tested recipient mice. This “positive control” setting was compared to minor-HAg mismatched GvH-HCT performed with MHC-congenic BALB.B recipients (group A2). A third parameter, however, complicates a direct comparison between the two types of recipients. From their B6 genetic background, C57BL/6 mice express Ly49H, an NK cell receptor that defines an NK cell subset present in C57BL/6 mice but missing in BALB.B mice. This is of relevance in the control of mCMV infection, as mCMV encodes the activatory Ly49H ligand m157 that induces a particularly strong NK cell response (Bubic et al., 2004). To exclude a putative contribution of residual Ly49H^+^ NK cells to the control of infection in C57BL/6 recipients, the deletion mutant mCMV-Δm157 was used to infect both types of recipients. The survival rates revealed a dramatic difference: all infected recipients of syngeneic HCT survived, whereas most infected recipients of H60 mismatched GvH-HCT died with a median survival time of 29 days.

In a second pair of HCTs (Figure 1B), we studied the impact of viral immune evasion on the clinical outcome of minor-HAg mismatched GvH-HCT. Immune evasion molecules, which are also referred to as “viral regulators of antigen presentation” (vRAPs; Holtappels et al., 2006), are m06/gp48 (Reusch et al., 1999; Fink et al., 2019) and m152 glycosylation isoforms p36 and gp40 (Ziegler et al., 1997; Fink et al., 2013). These vRAPs limit the presentation of antigenic peptides by interfering with cell surface trafficking of pMHC-I complexes (Lemmermann et al., 2010a, 2012). BALB.B recipients of GvH-HCT were infected with either WT mCMV expressing vRAPs (group B1) or with a virus mutant mCMV-ΔvRAP in which immune evasion genes *m06* and *m152* are deleted to enhance the cell surface presentation of pMHC-I complexes to CD8^+^ T cells (group B2). Notably, while significant lethality was observed after infection with WT virus, all recipients that were infected with the ΔvRAP mutant survived. Based on the somewhat reduced lethality from WT virus (group B1, Figure 1B) compared to Δm157 virus (group A2, Figure 1A) one might suppose that donor-derived Ly49H^+^ NK cells mediate some protection.

In conclusion so far, minor-HAg-mismatched GvH-HCT was associated with significant lethality that was prevented by enhancement of antigen presentation based on the deletion of vRAPs. Thus, in essence, this model reproduced key findings reported for the model of MHC class-I mismatched GvH-HCT (Holtappels et al., 2020). This is important as it indicates a more general mechanism that is not limited to MHC mismatch.

### Inquiry Into the Cause of Death in Infected Recipients of Minor-HAg-Mismatched GvH-HCT

Our work on MHC class-I-mismatched GvH-HCT has shown that lethal infection resulted from viral histopathology caused by an uncontrolled virus spread rather than from GvHD (Holtappels et al., 2020). It was therefore not far to seek that cytopathogenic infection of recipients' organs would also explain the lethality observed herein after H60-mismatched GvH-HCT.

**Figure 2:**
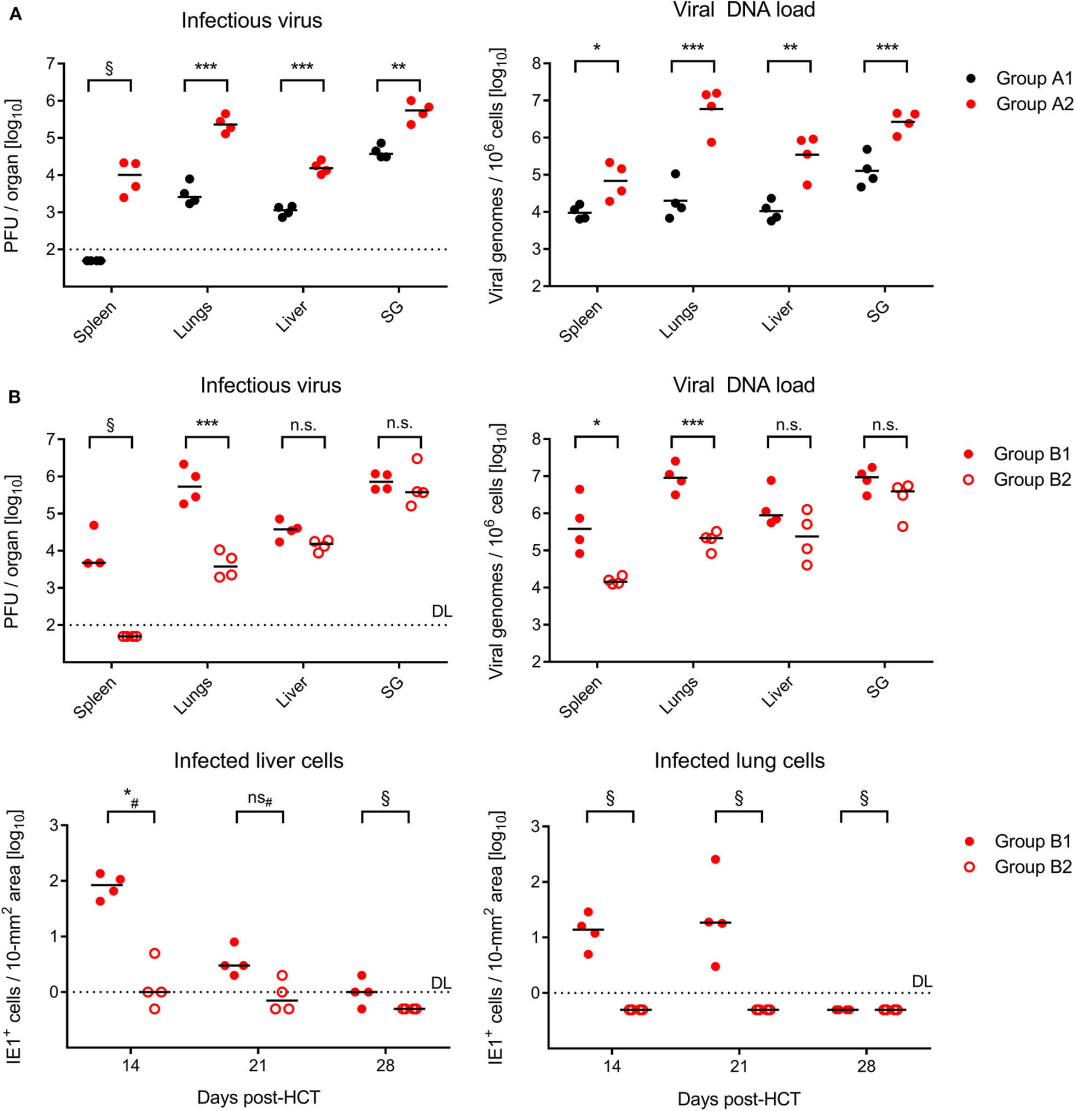
Control of organ infection in HCT recipients. **(A)** Quantitation of infectious virus (PFU, plaque-forming units; left panel) and viral DNA load (right panel) in organs of recipients of syngeneic HCT (group A1) and GvH-HCT (group A2) on day 21 after infection with Δm157 virus. SG, salivary glands. **(B)** *Upper panels*, quantitation of infectious virus and of viral DNA load on day 21 after GvH-HCT and infection with WT virus (group B1) or ΔvRAP virus (group B2). *Lower panels*, time course of infection control in liver (left panel) and lungs (right panel) determined by quantitation of infected tissue cells identified by IHC specific for the intranuclear viral protein IE1. Numbers of infected tissue cells refer to representative 10-mm^2^ areas of tissue sections. Note that data for day 28 are biased by unavoidable selection of survivors. Throughout, symbols represent mice analyzed individually. Median values are marked. DL, detection limit of the assay. Differences between two experimental groups were determined by Student's *t* test based on log-transformed log-normally distributed data. ^#^Distribution-free Wilcoxon-Mann-Whitney test for comparing groups with some values below DL. Significance levels: *P*-values of < 0.05 (^*^), < 0.01 (^**^), and < 0.001 (^***^). n.s., not significant. §, significance estimation not allowed and not reasonable, because one of the groups consists only of values below DL.

On day 21 after GvH-HCT and infection with Δm157 virus, that is at a time shortly before progression of disease leads to cases of death (recall Figure 1A), titers of infectious virus as well as viral genome loads were significantly elevated in GvH-HCT recipients (group A2) compared to recipients of syngeneic HCT (group A1) in all organs tested (Figure 2A). This finding clearly revealed a fundamental impact of minor-HAg mismatch on the control of virus spread in host tissues.

In the independent experiment for the second pair of HCTs (recall Figure 1B), day-21 titers of infectious virus as well as viral genome loads after infection with m157^+^ WT virus (group B1, Figure 2B) were as high as with the Δm157 mutant in all organs tested (group A2, Figure 2A). This finding argues against a notable role of donor-derived Ly49H^+^ NK cells in the control of virus spread. Accordingly, unlike we supposed above, they cannot account for the reduced lethality in group B1 compared to group A2 (recall Figure 1).

Notably, day-21 titers of infectious virus and viral genome loads were lower in GvH-HCT recipients infected with immune evasion gene deletion mutant ΔvRAP (group B2) compared to recipients infected with vRAP-expressing WT virus (group B1), although the difference was less pronounced and did not reach statistical significance in liver and salivary glands (Figure 2B). As a first ingenuous interpretation one may take this as evidence against the importance of liver infection for survival. One has to consider, however, that day-21 data already reflect an onset of immune control that may have come too late for the fraction of mice with later lethal outcome but obviously just in time for the fraction of survivors. We therefore quantitated infected cells in liver and lung tissue sections from recipients in groups B1 and B2 in the time course (Figure 2B, lower panels). These data clearly revealed a significantly better control of the immune evasion gene deletion mutant on day 14 at both of these pathogenetically most relevant organ sites, whereas the difference was increasingly leveled at later times. We thus conclude that an early control of liver and lung infection is critical for survival.

**Figure 3:**
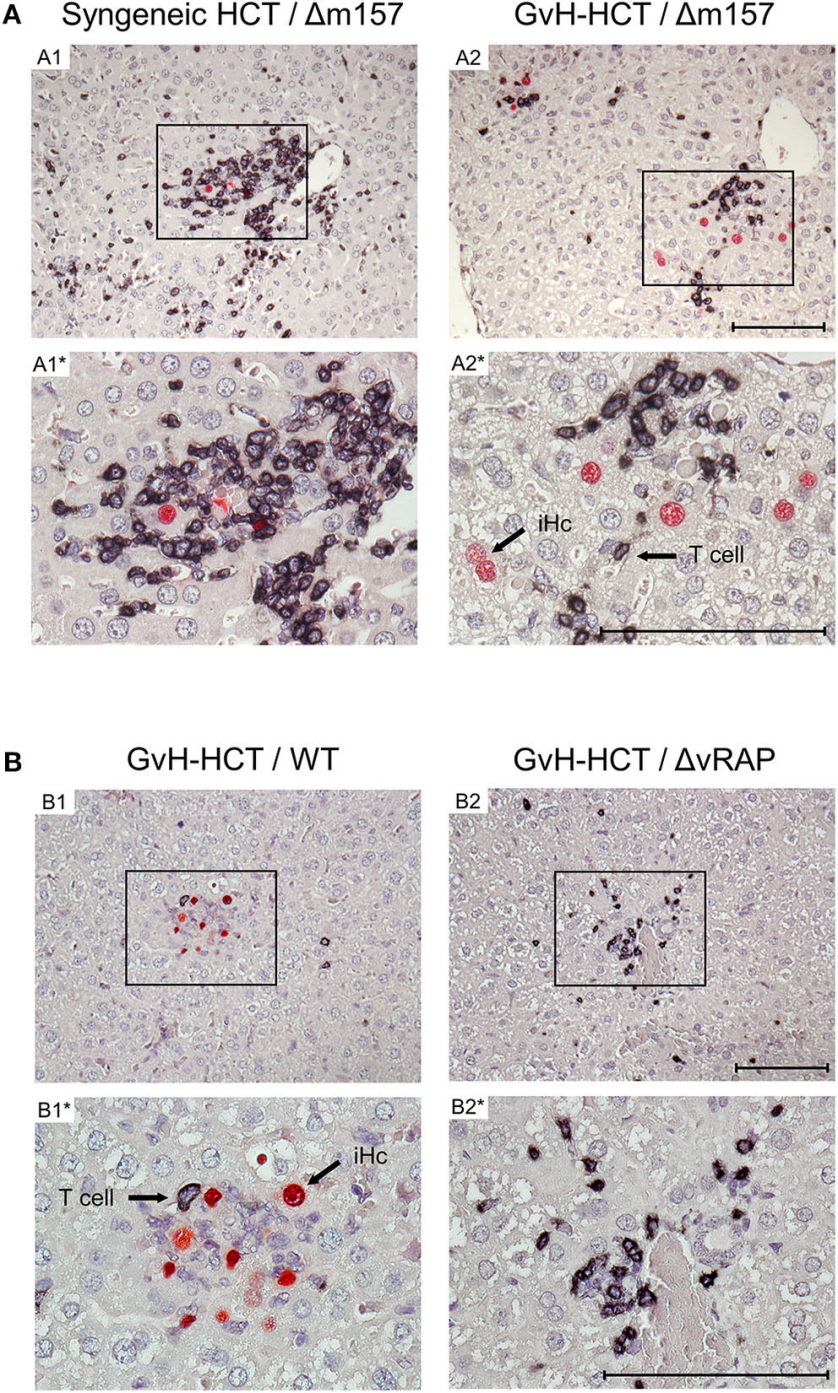
Immunohistological images of liver tissue infection and infiltration by T cells. **(A)** Comparison between syngeneic HCT (group A1) and minor-HAg mismatched GvH-HCT (group A2) on day 21 after infection with Δm157 virus. **(B)** Comparison of minor-HAg mismatched GvH-HCTs on day 14 after infection with WT virus (group B1) or ΔvRAP virus (group B2). Shown are representative 2C-IHC images with red staining of viral IE1 protein in nuclei of infected liver cells, which are mostly hepatocytes, and black staining of CD3ε protein expressed by T cells. Light counter-staining was done with hematoxylin. (A1, A2 and B1, B2), low magnification overview images. Frames in these overview images demarcate regions resolved to greater detail by higher magnification in images A1*, A2* and B1*, B2*, respectively. iHc, examples of infected IE1^+^ hepatocytes. Bar markers: 100 μm.

To relate virus spread or control to histopathological consequences, we show 2C-IHC images of tissue infection (red-stained intranuclear viral IE1 protein) and T-cell infiltration (black-stained CD3ε molecule) in liver and lung tissue sections (Figure 3, liver; Figure S1, lungs). As discussed in greater detail previously (Podlech et al., 2000; Böhm et al., 2008a; Holtappels et al., 2020; Renzaho et al., 2020), antiviral protection in tissues has a microanatomical correlate in the formation of nodular inflammatory foci (NIF). When virus-specific tissue-infiltrating CD8^+^ T cells recognize infected cells that display pMHC-I complexes at their cell surface (Böhm et al., 2008a; Lemmermann and Reddehase, 2016), they do not remain randomly distributed in tissue but congregate at infected tissue cells by forming NIF. By recognizing infected cells and by delivering effector functions, the NIF-localizing CD8^+^ T cells prevent further virus spread in tissue and eventually resolve productive infection.

Here, after syngeneic HCT and infection with Δm157 virus (group A1), CD3ε^+^ T-cell infiltrates, primarily consisting of CD8^+^ T cells as revealed by cytofluorometric analysis of isolated non-parenchymal liver cells, massively congregated at few remaining infected hepatocytes on day 21 shortly before onset of death in group A2 (recall Figure 1A). In contrast, after minor-HAg mismatched GvH-HCT and infection with Δm157 virus (group A2) clusters of tissue infiltrating CD3ε^+^ T cells also existed but seem to “ignore” the more frequent infected cells as they did not congregate there (Figure 3A). This indicates lack of antiviral control, likely because infected cells do not present antigen sufficient for recognition by the infiltrating cells. In accordance with this interpretation, on day 14 after GvH-HCT and infection with WT virus (group B1), at a time when differences were still prominent (recall Figure 2B), few liver-infiltrating T cells failed to form NIF and thus did not prevent virus spread that led to extended foci consisting of infected cells (Figure 3B, group B1). At the same time in the lungs, primordial NIF were formed but productive infection was not cleared yet (Figure S1, group B1). In contrast, in the absence of immune evasion after infection of GvH-HCT recipients with ΔvRAP virus, NIF were formed and had essentially cleared productive infection in the liver (Figure 3B, group B2) as well as in the lungs (Figure S1, group B2).

Interestingly, in an obvious discrepancy to our work on MHC class-I mismatched GvH-HCT (Holtappels et al., 2020), randomly distributed GvH-specific T cells recognizing the alloantigen on tissue cells were absent in liver and lungs (Figure 3 and Figure S1). This discrepancy is easily explained by the fact that MHC class-I molecules are expressed by all tissue cells, whereas the minor-HAg H60 is poorly expressed in the liver and not expressed at all in the lungs (Takada et al., 2008).

**Figure 4:**
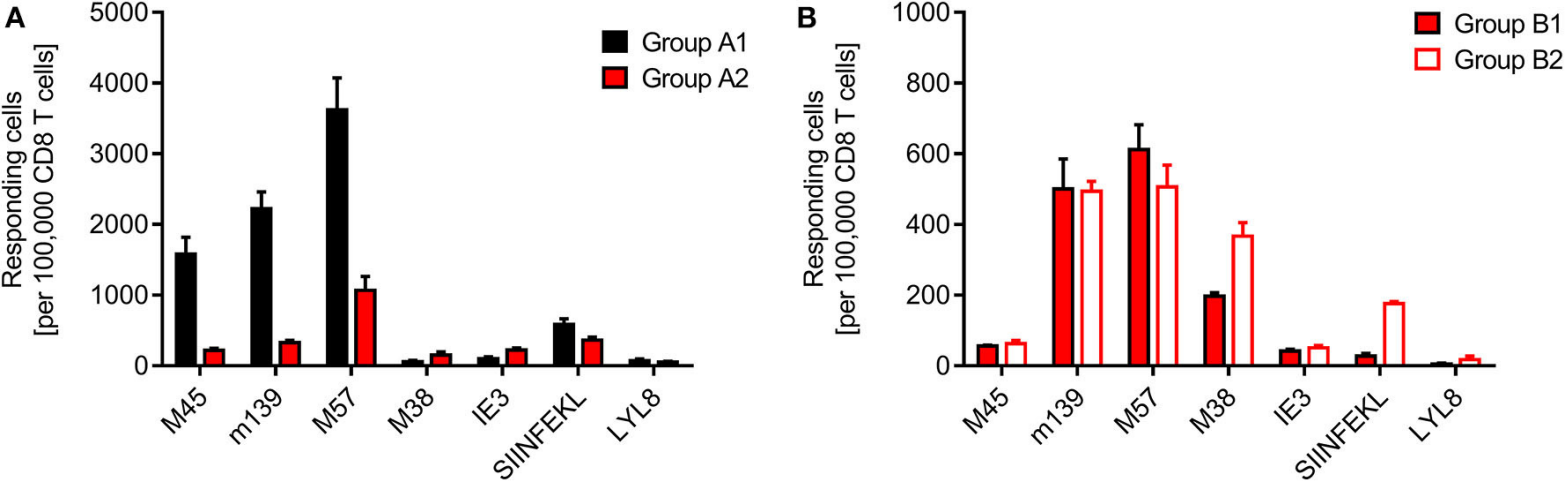
Quantitation of viral epitope-specific and minor-HAg H60-LYL8-specific responses. CD8^+^ T cells were isolated by immunomagnetic purification from spleens (yield from 5 pooled spleens in each group). **(A)** Comparison of syngeneic HCT (group A1) and minor-HAg mismatched GvH-HCT (group A2) on day 21 before onset of death cases in group A2 after infection with Δm157 virus. **(B)** Comparison of minor-HAg mismatched GvH-HCTs after infection with WT virus (group B1) or ΔvRAP virus (group B2) on day 21 before onset of death cases in group B1. Epitope-specific frequencies were determined by an ELISpot assay based on IFNγ secretion in response to stimulation with EL-4 (*H-2*^*b*^) target cells that were exogenously loaded with the indicated synthetic antigenic peptides at a saturating concentration of 10^−6^ M. Bars represent most probable numbers and error bars indicate the 95% confidence intervals determined by intercept-free linear regression analysis.

Finally, we determined the frequencies of viral and minor-HAg H60 epitope-specific CD8^+^ T cells in the spleen, comparing syngeneic HCT (group A1) with minor-HAg-mismatched GvH-HCT (group A2) (Figure 4A) as well as comparing minor-HAg-mismatched GvH-HCTs after infection with WT virus (group B1) and ΔvRAP virus (group B2) (Figure 4B). The spleen was chosen as a central lymphoid organ in which H60a is expressed, so that the epitope LYL8 should be presented at this site (Takada et al., 2008). The results clearly revealed a very poor reconstitution of epitope-specific functional IFNγ-secreting CD8^+^ T cells in GvH-HCT compared to efficient reconstitution after syngeneic HCT (Figure 4A). The comparison of GvH-HCTs after infection with WT virus (group B1) and ΔvRAP virus (group B2) confirmed the poor CD8^+^ T-cell response in the presence of immune evasion and added the information that the frequencies of responding cells were not substantially higher after infection with ΔvRAP virus (Figure 4B). They were actually not higher for viral epitopes M45, m139, M57, and IE3, though they were slightly elevated for the viral epitope M38 and the transgenic epitope SIINFEKL expressed by all 3 viruses used in this study. This suggests that the observed antiviral control in liver and lungs after vRAP deletion (Figure 2B and Figure 3B; Figure S1) was not based on higher numbers of primed virus-specific CD8^+^ T cells present in the spleen but on an improved antigen presentation by infected tissue cells.

At first glance, this finding of similar frequencies of epitope-specific CD8^+^ T cells in presence and absence of vRAPs appeared to contradict our previous finding of a much higher frequency of CD8^+^ T cells recognizing infected cells after infection of MHC class-I mismatched GvH-HCT recipients with ΔvRAP virus compared to WT virus (Holtappels et al., 2020). This, however, was found for liver-infiltrating CD8^+^ T cells and it was then left open if the observed increased frequency reflected a more efficient CD8^+^ T cell priming in lymphoid organs due to enhanced direct antigen presentation in the absence of vRAPs or reflected a more efficient recruitment of primed cells to non-lymphoid target organs of viral pathogenesis. In addition, we discussed a possible on-site proliferation after improved recognition of liver cells infected with ΔvRAP virus. Our current finding of similar frequencies in the spleen after infection with WT or ΔvRAP virus argues for the explanation that absence of vRAPs results in an enhanced tissue infiltration and possible on-site proliferation within protective NIF, eventually resulting in clearance of productive infection.

Although we have here not tested the functional avidity of the CD8^+^ T cells, data from the MHC-I mismatched GvH-HCT model (Holtappels et al., 2020) strongly suggest that the overall very low frequencies of epitope-reactive CD8^+^ T cells inherently imply that high-avidity cells, capable of recognizing infected cells also when antigen presentation is limited by immune evasion, are present at best in vanishingly low numbers. Notably, CD8^+^ T cells specific for the minor-HAg epitope LYL8 were undetectable in all three GvH-HCT groups (Figure 4, groups A2, B1, and B2), even though H60a, from which the LYL8 epitope can be processed, is expressed in the spleen. So, missing LYL8 presentation is unlikely the explanation for the missing response. We currently can only speculate that a GvH response, and accordingly GvHD, is prevented by transplantation tolerance, possibly involving regulatory T cells (Wood and Sakaguchi, 2003; Kang et al., 2007; Sykes, 2009).

Altogether, these findings for minor-HAg-mismatched GvH-HCT largely concur with the findings reported for MHC class-I-mismatched GvH-HCT (Holtappels et al., 2020), collectively arriving at the conclusion that lethal disease after GvH-HCT and mCMV infection does not represent GvHD but is caused by viral histopathology that results from inefficient reconstitution of functional antiviral CD8^+^ T cells.

As an outlook relevant for medical translation, our finding that lethality in GvH-HCT results from viral pathogenesis, rather than from GvHD, predicts that supplementing inefficient reconstitution of antiviral CD8^+^ T cells with transfer of mature, high-avidity virus-specific CD8^+^ effector T cells will protect against lethal infection in GvH-HCT recipients. In contrast, such an antiviral cytoimmunotherapy (Steffens et al., 1998a; Holtappels et al., 2008, 2013; Ebert et al., 2012; Renzaho et al., 2020) would obviously be no option to prevent histopathology from GvHD.

The question remains why an immunogenetic mismatch between donor and recipient leads to lethal viral disease despite a low (Holtappels et al., 2020) or absent (this report) mismatch-specific GvH response. Our previous discussion for the MHC class-I L^d^-mismatched GvH-HCT model (Holtappels et al., 2020) regarding missing presentation of antigenic peptides by L^d^ is now made pointless. Apparently, mismatch as such, rather than the type of the mismatch, determines the outcome of CMV infection in GvH-HCT recipients. As a perspective for future work, we put forward the hypothesis that any mismatches in transplantation antigens, be they MHC or minor-HAg mismatches, induce a “non-cognate transplantation tolerance” that dampens not only a mismatch-specific CD8^+^ T cell-mediated GvH-reaction, which is beneficial from a medical point of view, but fatefully also inhibits a protective response against pathogens like CMV.

The concordant finding of lethality from viral pathogenesis rather than from GvHD in the MHC-mismatched and minor-HAg-mismatched GvH-HCT mouse models of CMV infection predicts for clinical HCT that antiviral therapy is promising, whereas immunosuppressive therapy of GvHD may even be contraindicated.

## Data Availability Statement

The datasets generated for this study are available on request to the corresponding author.

## Ethics Statement

The animal study was reviewed and approved by the ethics committee of the Landesuntersuchungsamt Rheinland-Pfalz according to German federal law §8 Abs. 1 TierSchG (animal protection law), permission numbers 177-07/G10-1-052 and 177-07/G 14-1-015.

## Author Contributions

EG, JP, MR, and NL designed the study and are responsible for the analysis and interpretation of the data. EG, JP, KG, and SB conducted the work and analyzed the data. MR wrote the first draft of the manuscript. NL wrote sections of the manuscript. All authors contributed to manuscript revision, read, and approved the submitted version.

## Conflict of Interest

The authors declare that the research was conducted in the absence of any commercial or financial relationships that could be construed as a potential conflict of interest.

## Supplementary Material

Figure S1 Immunohistological images of lung tissue infection and infiltration by T cells depending on the expression of immune evasion genes. Representative 2C-IHC images of lung tissue sections are shown corresponding to the quantitation of infected lung cells on day 14 as shown in Figure 2B for minor-HAg-mismatched GvH-HCT groups B1 (infection with WT virus) and B2 (infection with ΔvRAP virus). (Red IHC staining) IE1 protein in nuclei of infected lung cells. (Black IHC staining) CD3ε protein expressed by T cells. Light counter-staining was done with hematoxylin. (B1 and B2) low magnification overview images. Frames in these overview images demarcate regions resolved to greater detail by higher magnification in images B1^*^ and B2^*^, respectively. Note that B1;B1^*^ show a peribrochiolar NIF. B2;B2^*^ show an interstitial NIF in the septum between alveoli. Bar markers: 100 μm.
