# Supplementary figures and images for: Enhancement of Antigen Presentation by Deletion of Viral Immune Evasion Genes Prevents Lethal Cytomegalovirus Disease in Minor Histocompatibility Antigen-Mismatched Hematopoietic Cell Transplantation
Source: Front Cell Infect Microbiol. 2020 Jun 9;10:279. doi: 10.3389/fcimb.2020.00279 (PMC7296086; doi:10.3389/fcimb.2020.00279)

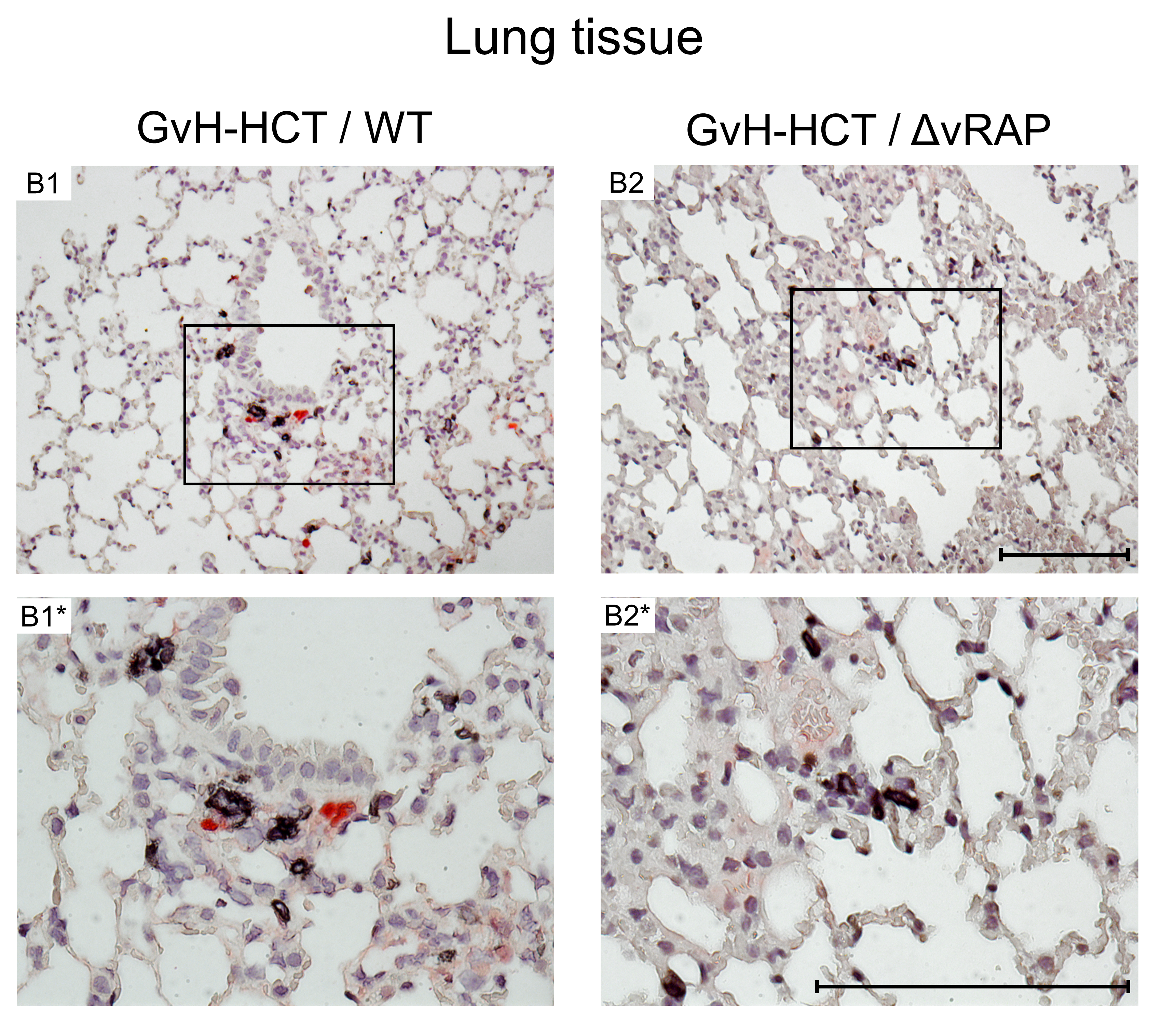

Supplement: Figure S1 — Immunohistological images of lung tissue infection and infiltration by T cells depending on the expression of immune evasion genes. Representative 2C-IHC images of lung tissue sections are shown corresponding to the quantitation of infected lung cells on day 14 as shown in Figure 2B for minor-HAg-mismatched GvH-HCT groups B1 (infection with WT virus) and B2 (infection with ΔvRAP virus). (Red IHC staining) IE1 protein in nuclei of infected lung cells. (Black IHC staining) CD3ε protein expressed by T cells. Light counter-staining was done with hematoxylin. (B1 and B2) low magnification overview images. Frames in these overview images demarcate regions resolved to greater detail by higher magnification in images B1* and B2*, respectively. Note that B1;B1* show a peribrochiolar NIF. B2;B2* show an interstitial NIF in the septum between alveoli. Bar markers: 100 μm. [file Image_1.TIF]
